# On recent developments in marginal separation theory

**DOI:** 10.1098/rsta.2013.0343

**Published:** 2014-07-28

**Authors:** S. Braun, S. Scheichl

**Affiliations:** Institute of Fluid Mechanics and Heat Transfer, Vienna University of Technology, Resselgasse 3, 1040 Wien, Austria

**Keywords:** laminar separation bubble, laminar–turbulent transition, viscous–inviscid interaction, triple-deck theory, adjoint operator method

## Abstract

Thin aerofoils are prone to localized flow separation at their leading edge if subjected to moderate angles of attack *α*. Although ‘laminar separation bubbles’ at first do not significantly alter the aerofoil performance, they tend to ‘burst’ if *α* is increased further or if perturbations acting upon the flow reach a certain intensity. This then either leads to global flow separation (stall) or triggers the laminar–turbulent transition process within the boundary layer flow. This paper addresses the asymptotic analysis of the early stages of the latter phenomenon in the limit as the characteristic Reynolds number 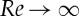, commonly referred to as marginal separation theory. A new approach based on the adjoint operator method is presented that enables the fundamental similarity laws of marginal separation theory to be derived and the analysis to be extended to higher order. Special emphasis is placed on the breakdown of the flow description, i.e. the formation of finite-time singularities (a manifestation of the bursting process), and on its resolution being based on asymptotic arguments. The passage to the subsequent triple-deck stage is described in detail, which is a prerequisite for carrying out a future numerical treatment of this stage in a proper way. Moreover, a composite asymptotic model is developed in order for the inherent ill-posedness of the Cauchy problems associated with the current flow description to be resolved.

## 1. Introduction

The investigation of laminar–turbulent boundary layer transition is of fundamental importance in respect of the understanding of the complicated structure of turbulence and also to develop appropriate engineering models for the prediction of flow characteristics. Of crucial theoretical as well as practical interest is the accurate calculation of lift and drag forces acting on aerodynamic bodies, which requires comprehensive knowledge on whether the flow is laminar or turbulent, attached or separated. The theory of boundary layer flows and, in particular, the examination of its most important issues, namely separation and transition when the Reynolds number is asymptotically large, have been a field of active research since Prandtl [1] presented his theory for laminar steady two-dimensional flows in 1904. Cornerstones of that development are, among others, the discovery of the singular behaviour and breakdown of the classical boundary layer equations near a point of vanishing skin friction (separation point in the case of steady flows) by Landau & Lifshitz [2] and Goldstein [3], and that of viscous–inviscid interaction independently made by Stewartson [4], Messiter 5 and Neiland [6] in the late 1960s, which has generally become known as the triple-deck theory. In conventional triple-deck problems, abrupt changes of boundary conditions or singular behaviour of the imposed pressure gradient initiate the interaction mechanism.

On the contrary, in cases of the so-called marginal separation, a (moderate) increase in the smooth imposed adverse pressure gradient controlled by a characteristic parameter leads to the onset of the interaction process and in further consequence to localized separation. In the early 1980s, Ruban [7,8] and Stewartson, Smith and Kaups [9] formulated a rational description of the local interaction mechanism now commonly referred to as the theory of marginal separation. It serves as the foundation of this work, which deals with the investigation of the early stages of the transition process triggered by the presence of laminar separation bubbles. As is well known, the theory of marginal separation predicts an upper bound of the control parameter for the existence of strictly steady, i.e. unperturbed, flows. The incorporation of unsteady effects led to the conclusion that the onset of the bursting process is associated either with exceeding the critical value of the control parameter or with the presence of a sufficient perturbation level in the case of below-critical conditions. Within the framework of the existing theory, vortex shedding from the rear of the separation bubble manifests itself in the occurrence of a finite-time singularity. Surprisingly, in that case recent findings strongly suggest the development of a unique blow-up pattern in leading order, entirely independent of the previous history of the flow [10]. The associated breakdown of the flow description implies the emergence of shorter scales, and the subsequent evolution of the flow then is described by a fully nonlinear triple-deck interaction, which seems to suffer finite-time breakdown as well [11,12]. The tracking of this ‘breakdown cascade’ is of particular interest and a main focus of the present investigation since it reflects the successive genesis of shorter spatio-temporal scales, which is a distinctive feature of the vortex generation process in transitional flows.

To highlight the main issues, we restrict ourselves to the most simple case of planar incompressible flows. Furthermore, it is assumed that the reader is familiar with the basic concept of marginal separation theory, which is well established. A very detailed description can be found in for example [13]. Our main focus is placed on the extension of the existing theory to higher orders (§2) for the purpose of formulating proper initial conditions for the triple-deck stage (§3) and addressing the observed ill-posedness of initial value problems associated with the current asymptotic flow description (§4).

## 2. Adjoint operator method

**Figure 1.**
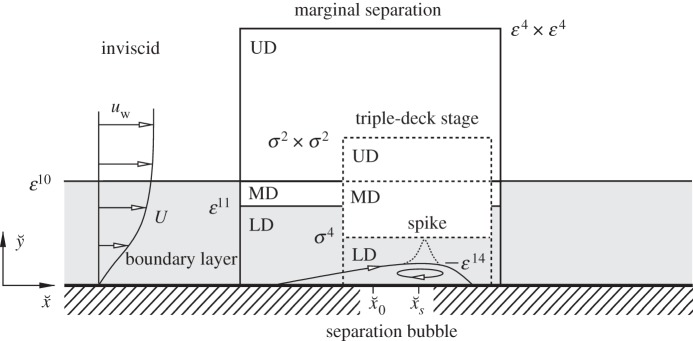
Asymptotic layer structure of laminar marginally separated flows including the phenomenon of bubble bursting (schematic). Spike formation initiated by a finite-time blow-up event at 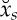, modelled up to the triple-deck stage (dashed lines): notation and order-of-magnitude relations in terms of the perturbation parameters *ε*=*Re*^−1/20^, *σ*=*Re*^−1/7^. Inviscid, irrotational upper decks (UDs), predominantly inviscid, rotational main decks (MDs), viscous boundary layer and lower deck (LD) regions (highlighted in grey).

In the following study, we reinvestigate the fundamental lower-deck (LD) problem of marginal separation theory. Here, however, we use an alternative method to that employed in the original studies [7,8,9,14] to derive the well-known similarity laws and their higher-order corrections, which govern the flow in the sublayer region LD (surrounded by a solid line in figure 1). To this end, non-dimensional quantities are introduced in the form
2.1
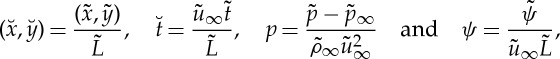
where 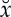 and 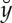 denote the coordinates in the streamwise direction and normal to the wall, *t* the time, *p* the pressure and *ψ* the stream function. The specific flow problem under consideration is characterized by the dimensional reference quantities: length 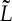, velocity 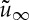, pressure 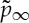, density 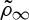 and kinematic viscosity 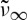, respectively (i.e. the unperturbed free stream values).

Particularly, we are interested in the behaviour of the boundary layer characteristics, i.e. displacement thickness 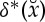 and wall shear stress 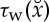,
2.2
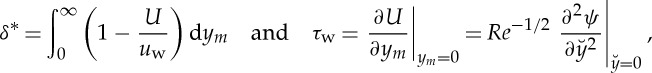
in the vicinity of a laminar separation bubble in the limit as the characteristic Reynolds number *Re* of the flow problem tends to infinity,
2.3
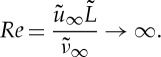
Here, 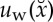 is the velocity of the outer inviscid flow at the solid wall, *y*_*m*_ the boundary layer (wall normal) coordinate and *U* the velocity distribution in the viscous boundary layer in the 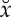-direction, which obeys the matching condition 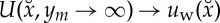. Furthermore, the usual scalings
2.4
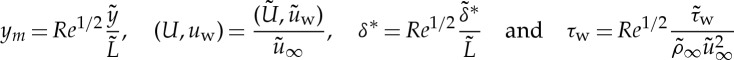
have been used.

The expansions of the stream function and the pressure gradient in terms of the perturbation parameter
2.5
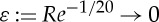
according to the original papers [7,8,9] for planar flow in the LD region in essence read
2.6
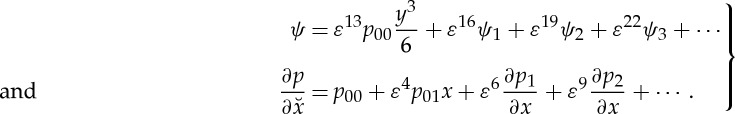
Here, the suitably scaled independent variables are denoted by
2.7
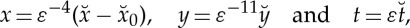
and the origin 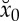 of the streamwise coordinate is chosen such that it coincides with the point where, according to classical boundary layer theory, the wall shear stress vanishes (and immediately recovers downstream) as the parameter *α* controlling separation attains its critical value *α*_c_. Furthermore, *p*_00_*y*^3^/6 represents the separation profile, where *p*_00_>0 is the imposed leading-order adverse pressure gradient; *ψ*_*n*_(*x*,*y*,*t*) and *p*_*n*_(*x*,*t*) characterize the perturbation stream functions and the induced pressures to be determined at the levels *n*=1,2,… of the approximation.

Substitution of (2.6) into the Navier–Stokes equations yields to leading order
2.8
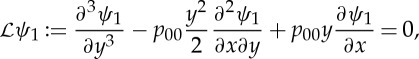
supplemented with the no-slip condition *ψ*_1_=∂*ψ*_1_/∂*y*=0 at *y*=0. In order to close the boundary value problem for (2.8), it is sufficient to require that *ψ*_1_ does not show exponential growth as 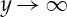. Then *ψ*_1_=*A*_1_*y*^2^/2+⋯ as 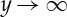 follows from (2.8), where the displacement function *A*_1_(*x*,*t*) remains arbitrary at this stage. As a consequence, the homogeneous solution (eigenfunction) of (2.8) is *ψ*_1_=*A*_1_*y*^2^/2. The function *A*_1_ is related to the displacement thickness and the wall shear stress (2.2) via the expansions
2.9
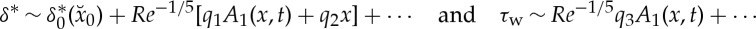
in the limit as 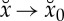 and *α*→*α*_c_. Here, 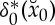 represents the value of the displacement thickness according to classical boundary layer theory in the limit *α*=*α*_c_ at 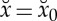, and the values of the constants *q*_1_<0, *q*_2_, *q*_3_>0 depend on the specific flow problem under consideration.

**Figure 2.**
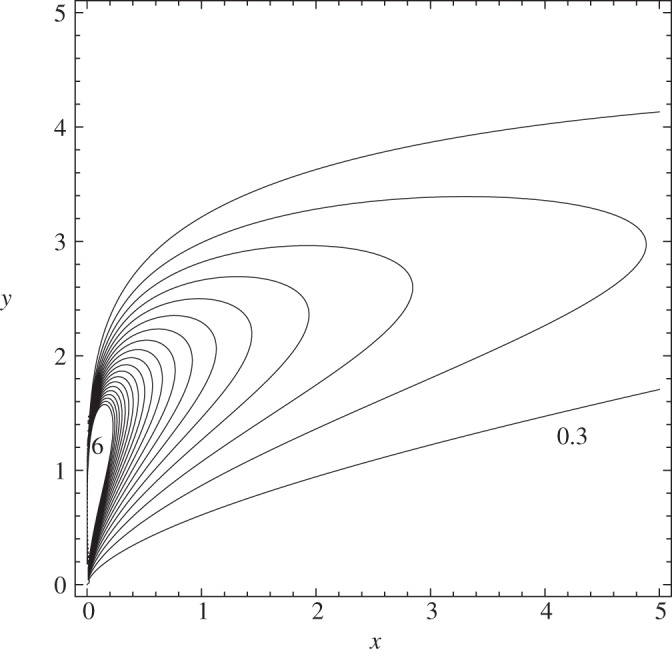
Contour plot of the eigenfunction *h*(*x*,*y*) of the adjoint to 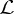, (2.21), indicating the strong decay in the *y*-direction, here with *p*_00_=1 in the range [0,6] and in increments of 0.3.

According to Fredholm's alternative, solutions of the inhomogeneous higher-order problems
2.10
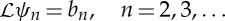
exist if and only if the right-hand sides *b*_*n*_ are orthogonal to the eigenfunction ℓ of the adjoint to 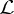 for the eigenvalue 0. This solvability condition (for *n*=2) determines *A*_1_ uniquely. To be specific, we multiply (2.8) with the yet unknown function ℓ(*x*,*y*) from the left and perform integration over the whole flow domain. Furthermore, we make use of the Fourier transform 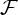 and Parseval's theorem for square-integrable functions *f* and *g* (a bar denotes the complex conjugate)
2.11
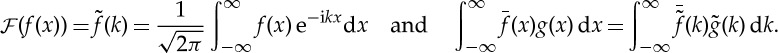
Then multiple application of integration by parts yields
2.12
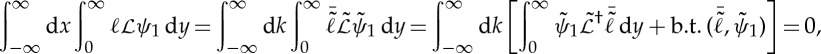
thus leading to the adjoint operator 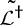 defined by
2.13
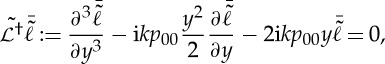
and the boundary terms
2.14
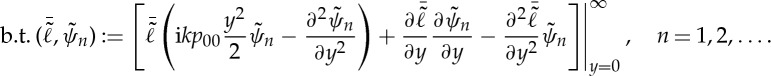
The general solution of (2.13) may be written as
2.15
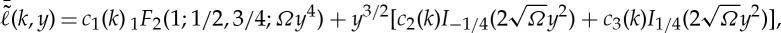
where _*p*_*F*_*q*_(*a*_1_,…,*a*_*p*_;*b*_1_…*b*_*q*_;*z*) denotes the generalized hypergeometric function, *I*_*ν*_(*z*) the modified Bessel function of the first kind and
2.16
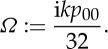
From the requirement of vanishing boundary terms 
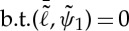
 in (2.12), we deduce the homogeneous boundary condition 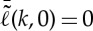 from (2.14), which leads to *c*_1_(*k*)=0. Furthermore, the suppression of exponential growth of 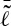 as 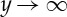 is ensured if *c*_3_(*k*)=−*c*_2_(*k*), which actually results in strong decay. Using the relation 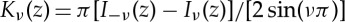 for the modified Bessel function of the second kind, one may write
2.17
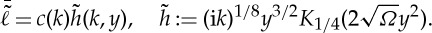
Application of the procedure underlying (2.12) to higher-order problems (2.10) results in
2.18
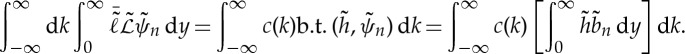
Since *c*(*k*) remains undetermined, we infer the solvability condition in Fourier space to be
2.19
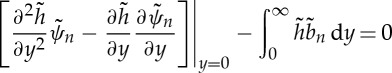
by using the explicit expression for the boundary term (2.14). Alternatively, inverse Fourier transform and application of the convolution theorem give the solvability condition in physical space as
2.20
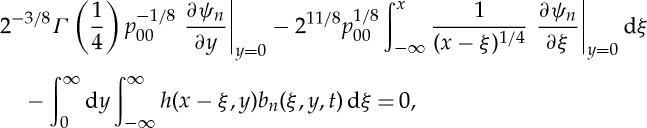
where
2.21
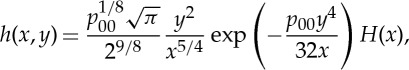
with *H*(*x*) denoting Heaviside's step function (figure 2).

The applicability of the procedure described above, in particular in respect of the use of the Fourier transform, requires sufficiently strong decay of *ψ*_*n*_ as 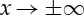. To ensure this prerequisite, a shift of the form
2.22
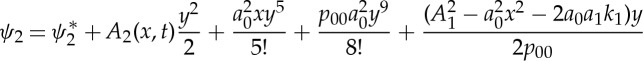
is performed, where *a*_0_,*a*_1_ are flow-problem-specific constants and *k*_1_=*ε*^−8^(*α*−*α*_*c*_)∼*O*(1) is the scaled control parameter; see [7,8] for details. From the matching to the up- and downstream boundary layer region, it is known that *A*_1_∼*a*_0_|*x*|+*a*_1_*k*_1_/|*x*|+⋯ as 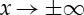; this far-field behaviour can be deduced from a local analysis of the classical (non-interactive) boundary layer equations near a point of vanishing skin friction [7]. The corresponding problem now reads
2.23
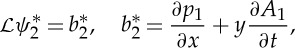
with the modified no-slip condition 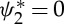, 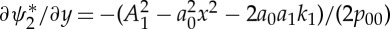 at *y*=0. From (2.20) with the use of (2.21) and
2.24
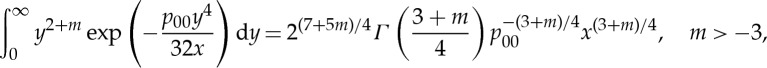
one immediately obtains—without the necessity to know the solution 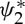
—the well-known fundamental equation for *A*_1_,
2.25
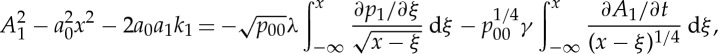
with the abbreviations
2.26
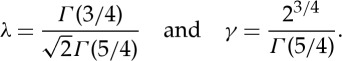
See [8,9] for the steady and [11,15] for the unsteady flow case. For the incompressible flow case considered here, (2.25) has to be supplemented with the upper-deck (UD) solution, i.e. the interaction law
2.27
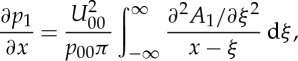
where *U*_00_ is the velocity at the outer edge of the boundary layer evaluated at the separation point *x*=0.

Self-evidently, (2.19) can also be evaluated. Taking into account (2.17) and
2.28
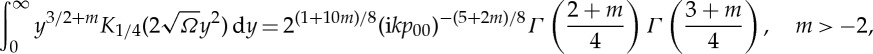
one then obtains
2.29
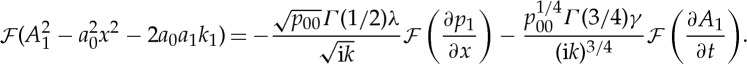
The Fourier transform of the pressure gradient (2.27) may be written as
2.30
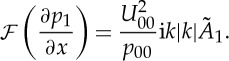
By applying the Fourier transforms of the Weyl fractional integrals
2.31
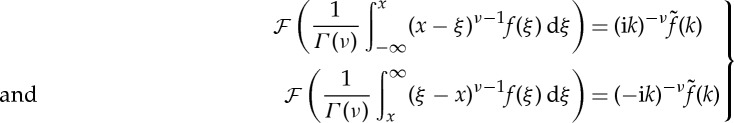
with the restriction 0<*ν*<1, one immediately recovers (2.25). Commonly, affine transformations are introduced to eliminate the problem-specific constants *a*_0_,*a*_1_,*p*_00_,*U*_00_ and to underline the similarity law character of the fundamental equation (2.25) in combination with (2.27). With respect to the passage of the ‘marginal separation stage’ into the triple-deck stage (via finite-time blow-up), we prefer to keep these constants in the corresponding equations.

Moreover, it should be emphasized that the effects of flow control elements (‘smart structures’), such as surface-mounted obstacles and/or suction/blowing devices, can easily be incorporated into the analysis, for the latter, e.g. the second term in (2.20), does not vanish. The additional terms thus resulting may also be used for the formulation of appropriate initial value problems, as studied in [10] or §4.

We are now in the position to extend the theory of marginal separation to second order. To this end, the explicit solution 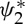 of (2.23) is required, which is known in closed form in Fourier space only and may be found by means of a power series ansatz [13]:
2.32
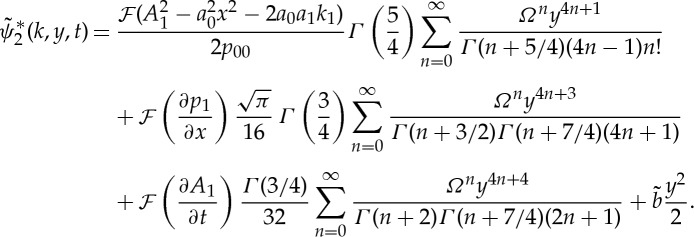
The arbitrary function 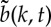 is chosen such that 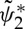 does not grow algebraically ∼*O*(*y*^2^) as 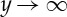, and, in combination with (2.29), one can alternatively rewrite (2.32) in terms of generalized hypergeometric functions:
2.33
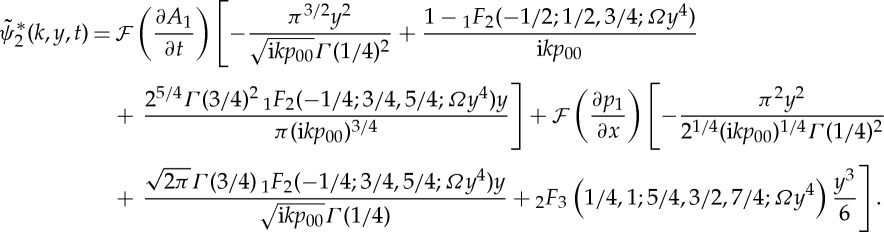
The far-field behaviour of 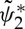 and its contribution to the wall shear stress are consequently given by
2.34
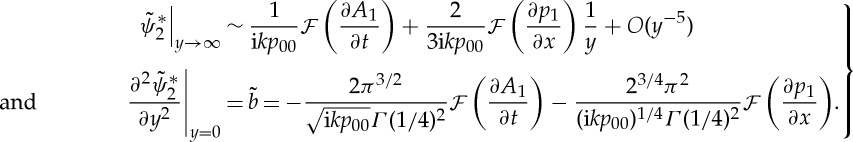
In order to determine the second-order correction displacement function *A*_2_, we follow the procedure described above and introduce, similar to (2.22), the shift
2.35
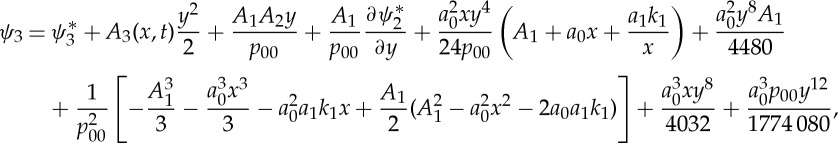
which leads to
2.36
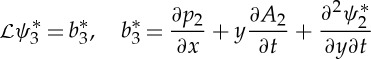
and the boundary conditions
2.37
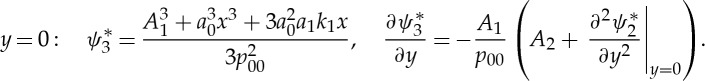
Substitution of the Fourier-transformed versions of (2.36) and (2.37) into the solvability condition (2.19), evaluation by means of (2.32), (2.34) and (2.28), and application of the inverse Fourier transform yields the forced linear, fundamental equation for *A*_2_:
2.38
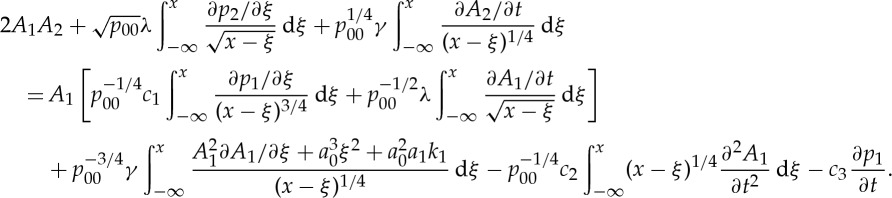
Here
2.39
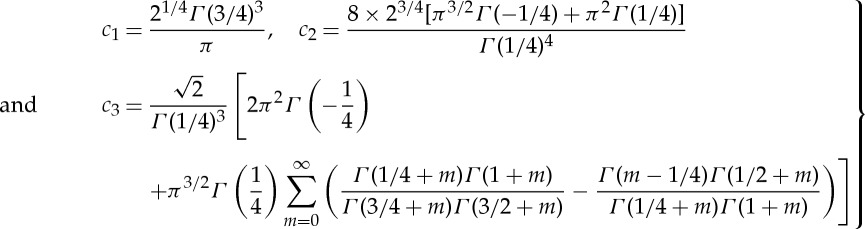
are positive constants (*c*_2_≈0.661 009, *c*_3_≈0.108 380). For the closure of (2.38), in addition to (2.27) a relationship between *p*_2_ and *A*_2_ is required. Investigation of the UD region (figure 1) leads to
2.40
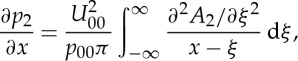
see (4.15). An investigation similar to that performed in the appendix of [10] yields the far-field behaviour *A*_2_∼*O*(|*x*|^−3/4^) as 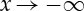 and *A*_2_∼*O*(*x*^7/4^) as 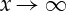.

## 3. Finite-time blow-up and the subsequent triple-deck stage

**Figure 3.**
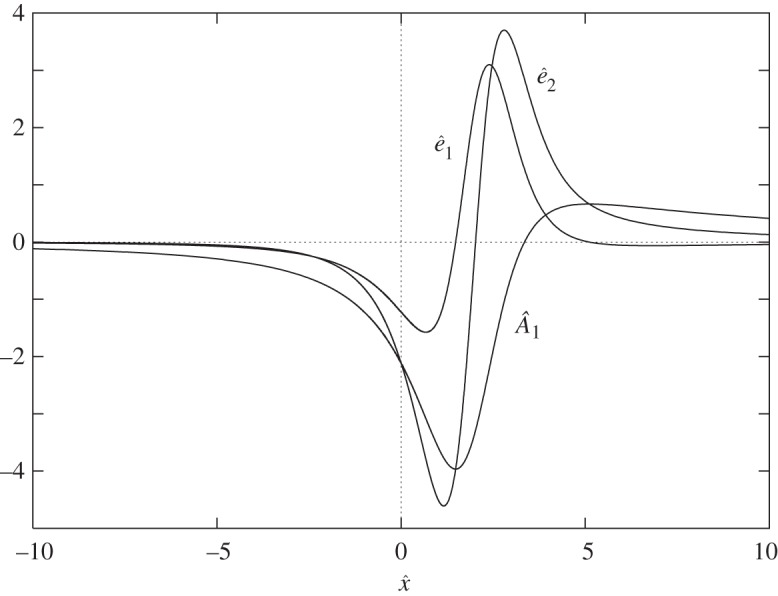
Unique blow-up profile 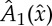 according to (3.12) and eigenfunctions 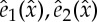, equations (3.13) or (3.14), for the special choice *p*_00_=1.

As is known from [11], solutions of the fundamental equation for *A*_1_ may blow up at a finite time *t*_*s*_, at a single point *x*_*s*_ under certain conditions (e.g. sufficiently strong forcing for below-critical control parameter conditions or above-critical conditions even without any forcing, e.g. [16]). The specific behaviour is given by
3.1
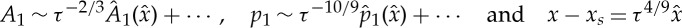
as *τ*:=*t*_*s*_−*t*→0^+^. Recent investigations show that the blow-up profile 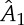 or, equivalently, 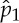 is unique [10] (figure 3), and consequently the following question arises. How do the initial conditions, effects of flow control devices, etc. (the ‘history’ of the flow) enter the matching condition to the subsequent fully nonlinear triple-deck stage first studied in [11,12] and sketched in figure 1? With the appropriate rescalings in the LD region
3.2
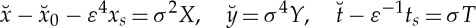

and
3.3
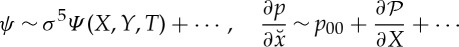
as *σ*:=*Re*^−1/7^→0, one obtains, after substitution into the Navier–Stokes equations, the fundamental LD problem
3.4
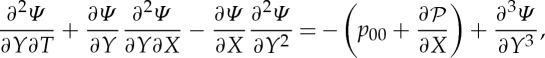
subject to the no-slip boundary conditions *Ψ*=∂*Ψ*/∂*Y* =0 at *Y* =0 and the far-field behaviour
3.5
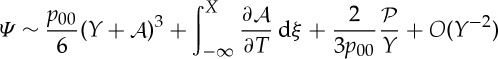
as 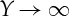 and 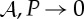 as 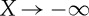. In addition, the interaction law connecting the displacement function 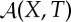 and the induced pressure 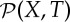 in the UD for incompressible flows
3.6
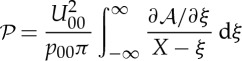
is recovered. As expected, the local displacement effect and the action of the wall shear stress (2.2) become intensified
3.7
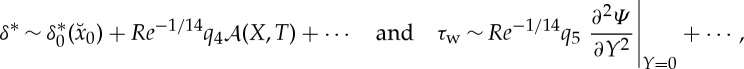
cf. (2.9). Here *q*_4_<0, *q*_5_>0 again denote problem-specific constants.

Combining expansions (2.6) and the (blow-up) scalings (3.1)–(3.3) results in the initial/matching condition as 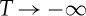, *τ*→0^+^:
3.8
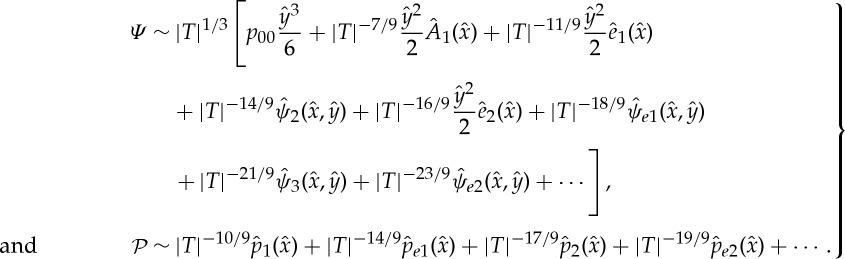
Here, 
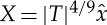
, 
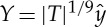
, and 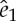, 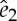 denote eigenfunctions resulting from the ansatz 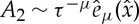 for the homogeneous part of (2.38) with eigenvalues *μ*=(10/9,15/9), respectively. Furthermore, 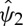 represents the limiting solution *ψ*_2_ as *τ*→0 and, similar to (2.22), we introduce
3.9
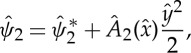
with a yet unknown correction displacement function 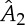, which enters the asymptotic representation of the triple-deck displacement function
3.10
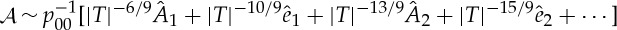
as 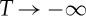. An asymptotic expansion of (3.4) based on (3.8) immediately leads to
3.11
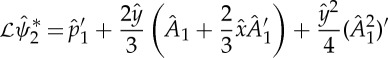
with 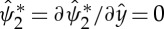 at 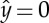. The solvability condition (2.20) then yields the equation for the blow-up profile
3.12
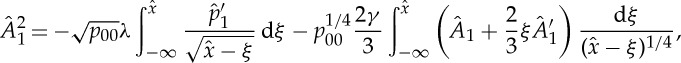
where 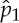 and 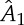 are related via (3.6) (figure 3). Similarly, we obtain
3.13
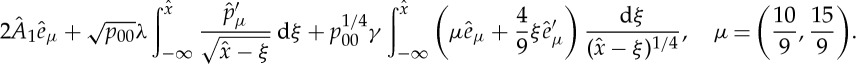
Here, the indeterminate amplitudes of 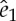 and 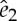 carry the ‘history’ of the flow and may be converted into a shift of the blow-up point *x*_*s*_→*x*_*s*_+Δ*x*_*s*_ and *t*_*s*_→*t*_*s*_+Δ*t*_*s*_ with Δ*x*_*s*_,Δ*t*_*s*_≪1, respectively (formulation (3.4) is invariant with respect to a shift in *X* and *T*). Furthermore, the shapes of 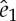 and 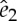 correspond to *x* and *t* derivatives of *A*_1_ in the limit as *τ*→0 (figure 3)
3.14
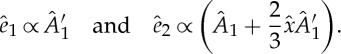
To determine the blow-up profile 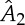, analogously to (2.35), 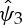 is written as
3.15
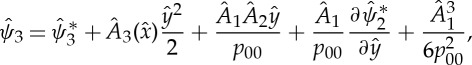
which, on further expansion of (3.4), leads to
3.16
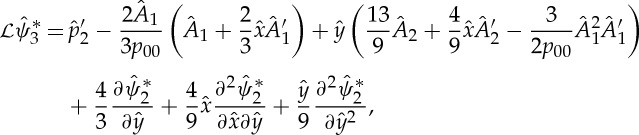
supplemented with the modified no-slip boundary conditions
3.17
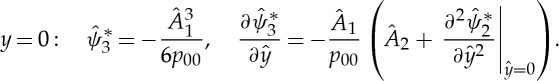
Application of the procedure that led to (2.38) here then yields
3.18
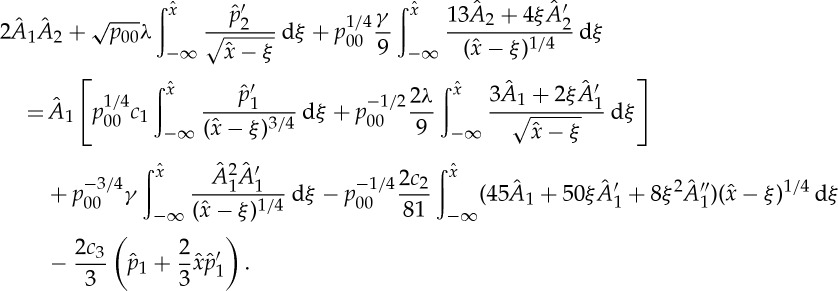
Alternatively, one can derive (3.18) from (2.38) by taking the limit *t*→*t*_*s*_ and using the blow-up scalings (3.1), (3.8) and (3.10).

With the blow-up profiles 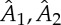, the eigenfunctions 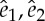 (with arbitrary amplitudes) and the stream function 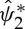 determined numerically, we are able to properly start the triple-deck computations. This involved task is currently under investigation.

In general, the Cauchy problems associated with the fundamental problem (2.25) and (2.27) and the triple-deck stage (3.4)–(3.8) are known to be ill-posed [17,12], but can be regularized if the streamline curvature is taken into account; see [18–20] and the following section for a detailed analysis.

## 4. Regularization terms

In a recent study by the present authors [10], initial value problems based on (2.25) together with (2.27), i.e.
4.1
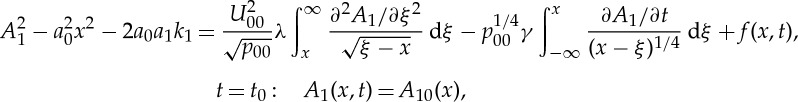
were addressed numerically. Here, the additional term *f*(*x*,*t*) accounts for the forcing due to control devices such as a surface-mounted obstacle or a suction slot, respectively, and *A*_10_(*x*) is the solution to the steady and unforced version of (4.1). Numerical solutions to the steady problem for different values of the parameters *k*_1_, *a*_0_, *a*_1_, *p*_00_ and *U*_00_, which by application of an affine transformation to (4.1) can be combined into a single control parameter, are presented, for example, in the original works [8,9]. In the analysis [10], special emphasis was placed on solutions that terminate in the form of finite-time singularities, and comprehensive numerical computations convincingly demonstrated the development of a unique blow-up structure, entirely independent of the particular choice of initial data. As already outlined before, further support for this important result was provided by an asymptotic analysis of (4.1) near the blow-up point, where the resulting equation admits a unique solution that is in perfect agreement with the numerical findings.

On the other hand, some inconsistencies associated with the proposed Cauchy problem (4.1) were discovered in [15], and their repercussions on the solvability in general are discussed in detail in [17]. Both works mention the occurrence of instabilities in the high-wavenumber regime, with the latter, however, putting this in the context of an ill-posed Cauchy problem. More precisely, it was shown that the absolute instability of the velocity field against short-scale disturbances and, entailed by that, the incorrectness of the problem essentially are a direct consequence of the abnormal dispersion relation governing the linearized problem in the limit of very high wavenumbers. Using a Fourier approach in the sense of a global stability analysis such that
4.2
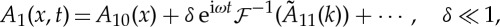
where *k* is real, one can derive from (2.29) and (2.26) the asymptotic result
4.3
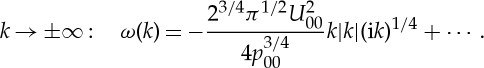
This yields that, to leading order in the high-wavenumber limit, the left-hand side of (2.29) does not affect the dispersion relation and, above all, that the growth rates for short-scale instabilities
4.4
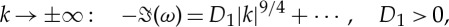
are not bounded from above. Therefore, the Cauchy problem as given in (4.1) turns out to be ill-posed and has to be regularized.

The essence of the above discussion is that higher-order terms in *ε* not included so far must play a distinctive role in the evolution of the unsteady marginal separation process, given the bounded spectrum that the unsteady Navier–Stokes equations are assumed to have, but which the leading-order problem fails to deliver. In the light of this, the absence of short-scale instabilities in the numerical solutions presented in [10] has to be attributed to the applied (semi-)implicit scheme. Obviously, the small but, nonetheless, always present terms that result from the discretization and that are of the order of the truncation error proved very effective in regularizing the problem. For the same reason, the steady solutions serving as initial conditions had to be destabilized by sufficiently strong forcing in order for the blow-up to be triggered. On physical grounds, however, the unrealistic growth of self-excited waves should be avoided by analysing the Navier–Stokes equations up to higher orders rather than implementing a numerical scheme designed for producing the desired results. In anticipation of what follows, the solutions presented in [10] will nevertheless turn out to be consistent with the then well-posed problem.

To the best of the authors' knowledge, there does not exist a rigorous asymptotic theory for the regularization of otherwise ill-posed Cauchy problems. The error introduced by omitting higher-order terms of the original Navier–Stokes equations falls beyond the scope of an asymptotic leading-order approach. In other words, the filtering process entailed by the temporal and spatial scales on which the asymptotic analysis is based may lead to the undesired result that the terms needed for regularizing the Cauchy problem at a certain level of approximation cannot be incorporated into it. They will thus enter the equations of higher order and, by that, form the higher-order forcing terms. Thus, the only way to regularize the original problem is to set up a *composite* asymptotic model, where terms of higher order are taken as part of the leading-order problem such that they can effectively contribute to stabilizing its homogeneous solution against short-scale disturbances.

This method has proved to be very successful in the past (e.g. [21,22,19] and references therein) and will also be applied here. As revealed in these studies, for higher-order asymptotic terms to play the role of regularization terms, they must at least generate *derivatives* with respect to *t* and *x* of orders that are higher than those already present in the system. Inspection of the inhomogeneous terms in equation (2.38) for *A*_2_ when they are considered as part of the leading-order problem shows that, although they then do represent derivatives of higher order, essentially the same high-wavenumber limit (4.3), merely multiplied with a positive factor, is obtained. The expression for the growth rates (4.4) thus remains unchanged. However, the pressure induced by the second-order displacement and, to even higher order in *ε*, by the LD stream function *ψ*_2_ has not been examined until now, and from the arguments put forward in [21,22] it can be deduced that precisely the higher-order terms associated with *ψ*_2_ as 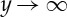 will give rise to physical phenomena that have a beneficial effect on the regularization of the leading-order Cauchy problem.

In the main-deck (MD) region of the marginal separation stage, where 
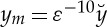
, the expansion of the stream function resulting from the LD solutions *ψ*_1_ and *ψ*_2_ as 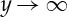, see (2.34), thus assumes the form
4.5
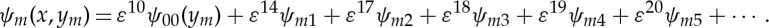
Substitution of (4.5) into the horizontal momentum equation then yields to leading order
4.6
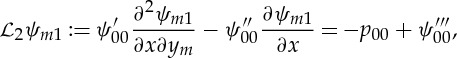
and additionally,
4.7
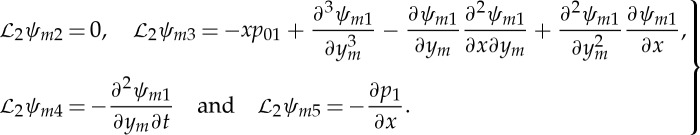
The last equation in (4.7) is interesting insofar as that here the induced pressure component of leading order *O*(*ε*^10^) enters. Furthermore, in the main layer, 
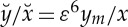
 and the pressure induced by *ψ*_*m*5_ in the UD and acting in the LD forms the component of *O*(*ε*^16^) in the pressure expansion. These facts suggest a pressure variation in the *vertical* direction for precisely this term of *O*(*ε*^16^) to come into play, since its normal gradient will be comparable in size with the leading-order convective term in the vertical momentum equation. Consequently, the extended version of the *horizontal* pressure gradient in the MD is given by
4.8
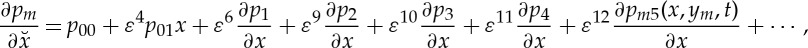
where, as in (2.6), all terms representing Taylor expansions of orders higher than *O*(*ε*^4^) have been omitted since they will not contribute to the results presented in the following. Most important, all terms in this expansion, except for *p*_*m*5_(*x*,*y*_*m*_,*t*), are identical to the corresponding components in the LD. As already indicated by the above order-of-magnitude argument, the asymptotic expansion of the vertical momentum equation then leads to the relationship
4.9
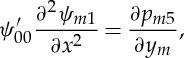
revealing the intrusion of a normal pressure gradient. As will be shown in the following, the last equation in (4.7) together with (4.9) will induce a pressure response in the LD that is proportional to the curvature of the streamlines, i.e. the second derivative of the displacement function *A*_1_ with respect to the streamwise coordinate.

After applying the rules for matching with the LD and extracting the singular parts of the resulting integrals, one can write the solutions to (4.6) and (4.7) as
4.10
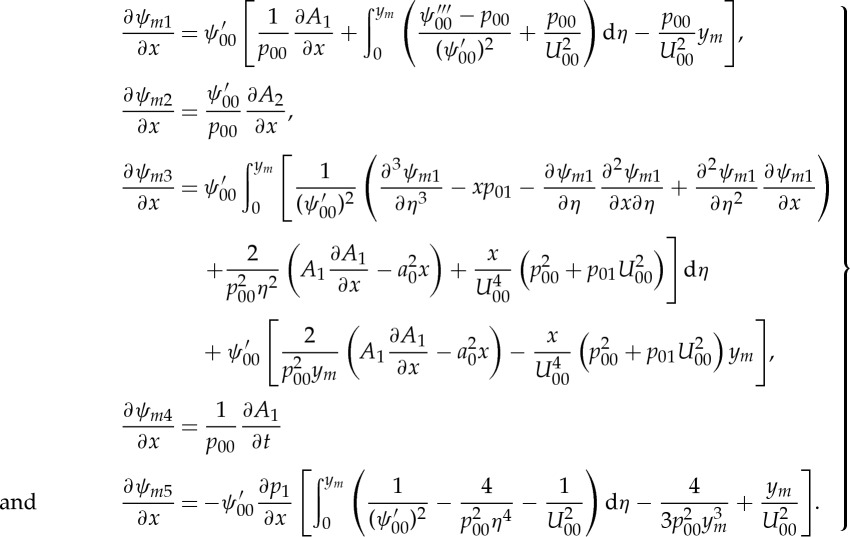
Note that for the derivation of these expressions, the expansions *y*_*m*_→0: 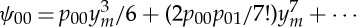 and 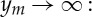 *ψ*_00_=*U*_00_*y*_*m*_+⋯ have been used (e.g. [9]). In addition, the equation for the normal pressure gradient (4.9) results in the expression
4.11
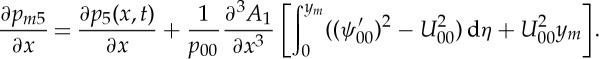
Here, ∂*p*_5_/∂*x* denotes the induced pressure gradient of order *O*(*ε*^12^) acting in the LD, see (2.6), which remains to be determined later.

As mentioned above, the purpose of the analysis presented here is the identification of those pressure terms induced by the leading-order displacement function *A*_1_ that are promising candidates for regularizing the Cauchy problem stated in (4.1). Therefore, and for simplicity, LD displacement effects of orders higher in *ε* than that represented by *A*_2_ have been omitted in the MD solutions. For the same reason, furthermore, the pressure generated by the component *ψ*_*m*3_ will not be considered in the following, since neither the linear nor the quadratic nonlinear terms contained in *ψ*_*m*3_ will lead to *derivatives* of *A*_1_ with respect to *x* or *t* that are of higher order than that already present in the leading-order term *ψ*_*m*1_.

The thus simplified UD expansions, with the appropriate coordinate normal to the wall now being 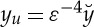, are
4.12
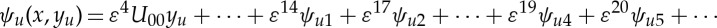
and
4.13
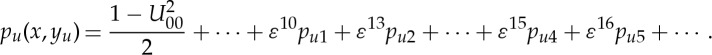


Through substitution into the momentum equations, it is easy to verify that the components of the stream function and the pressure satisfy
4.14
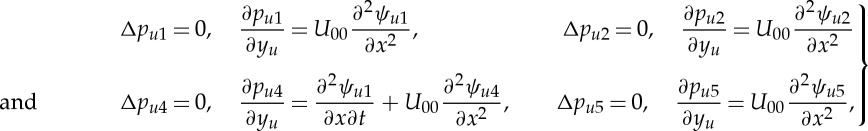
where Δ is the Laplacian 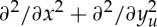. Relying on the matching principles and taking into account that apart from *p*_*m*5_ the MD pressure terms do not depend on *y*_*m*_, one then obtains from (4.14) and (4.10)
4.15
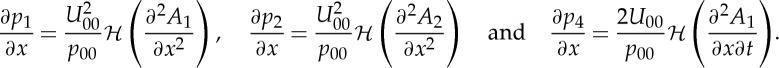
Here, 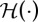 denotes the Hilbert transform, i.e.
4.16
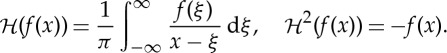
Consequently, the well-known leading-order result (2.27) for *p*_1_ is recovered and also the problem for *A*_2_ as given in (2.38) can now be stated in closed form. Furthermore, it should be noted here that, as a matter of course, the singular parts of the solutions (4.10) as 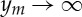 match seamlessly with the corresponding counterparts in the UD. Moreover, the last equations in (4.14) and (4.10), respectively, together with (4.11) lead to
4.17
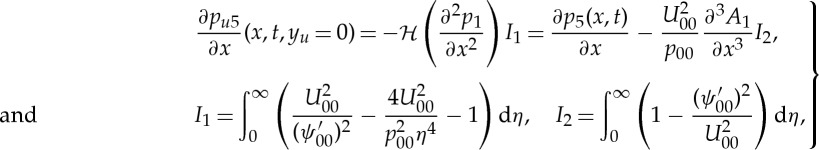
and from (4.15) and (4.16), the LD pressure gradient ∂*p*_5_/∂*x* is inferred to be given by
4.18
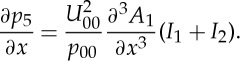
Interestingly, results very similar to this and that for ∂*p*_4_/∂*x* were found in the triple-deck analyses of the Blasius boundary layer with respect to the so-called lower branch stability [23] and, respectively, the associated acoustic radiation [24].

In the following, it will be assumed that *ψ*_00_′ is smaller than *U*_00_ throughout the MD such that both *I*_1_ and *I*_2_ are positive quantities. As a consequence, the induced pressure gradient in the LD replacing ∂*p*_1_/∂*x* from (2.27) in order to form a *composite* model equation when introduced into (2.25) can be written as
4.19
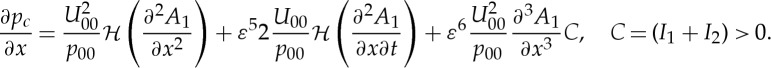
Accordingly, the modified dispersion relation derived from (2.29) for the compound model reads
4.20
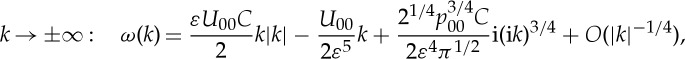
leading to
4.21
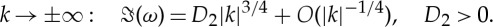
From this, it can be concluded that the growth rates −ℑ(*ω*) are bounded from above and tend to 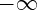 in the limit of very high wavenumbers, which, in turn, gives good reason to assume that the Cauchy problems based on this corrected model are well-posed.

In order to test this assumption, we re-examined the numerical solutions presented in [10] for a different initial value problem by using the extended model for the pressure gradient (4.19). In all cases, the temporal evolution of the leading-order displacement function *A*_1_ as well as the development of the blow-up structure could be recalculated without difficulties. The results perfectly agree with those already given in that paper, even when other numerical schemes were applied that definitely would have failed due to the lack of inherent numerical regularization terms if the uncorrected model had been used.

## 5. Conclusion

This paper addresses an adjoint operator approach to the calculation of higher-order displacement effects in marginal separation theory. In further consequence, this method is used to determine the initial conditions necessary for a proper formulation of the triple-deck stage, which is initiated due to a finite-time blow-up event in the marginal separation stage. Moreover, it is shown that the application of a composite asymptotic model which accounts for higher-order effects, such as streamline curvature, successfully leads to a regularization of the ill-posedness associated with initial value problems in marginal separation theory.

## References

[1] Prandtl L 1905 Über Flüssigkeitsbewegung bei sehr kleiner Reibung. Verhandlungen des III Internationalen Mathematiker-Kongress, Heidelberg, 1904, pp. 484–491 Leipzig, Germany: Teubner.

[2] Landau LD Lifshitz EM 1944 Mechanics of continuous media Moscow, Russia: Gostekhizdat.

[3] Goldstein S 1948 On laminar boundary-layer flow near a position of separation. Q. J. Mech. Appl. Math 1, 43–69. (10.1093/qjmam/1.1.43)

[4] Stewartson K 1969 On the flow near the trailing edge of a flat plate II. Mathematika 16, 106–121. (10.1112/S0025579300004678)

[5] Messiter AF 1970 Boundary-layer flow near the trailing edge of a flat plate. SIAM J. Appl. Math. 18, 241–257. (10.1137/0118020)

[6] Neiland VYa 1969 Theory of laminar boundary layer separation in supersonic flow. Izv. Akad. Nauk SSSR 4, 53–57. (10.1007/BF01094681)

[7] Ruban AI 1981 Singular solution of boundary layer equations which can be extended continuously through the point of zero surface friction. Izv. Akad. Nauk SSSR 6, 42–52. (10.1007/BF01089710)

[8] Ruban AI 1982 Asymptotic theory of short separation regions on the leading edge of a slender airfoil. Izv. Akad. Nauk SSSR 1, 42–51. (10.1007/BF01090696)

[9] Stewartson K Smith FT Kaups K 1982 Marginal separation. Stud. Appl. Maths. 67, 45–61.

[10] Scheichl S Braun S Kluwick A 2008 On a similarity solution in the theory of unsteady marginal separation. Acta Mech. 201, 153–170. (10.1007/s00707-008-0079-6)

[11] Smith FT 1982 Concerning dynamic stall. Aero. Q. 33, 331–352.

[12] Elliott JW Smith FT 1987 Dynamic stall due to unsteady marginal separation. J. Fluid Mech. 179, 489–512. (10.1017/S0022112087001629)

[13] Ruban AI 2010 Asymptotic theory of separated flows. In Asymptotic methods in fluid mechanics: survey and recent advances (ed. Steinrück H). CISM Courses and Lectures, vol. 523, pp. 311–408. Wien New York, NY: Springer.

[14] Stewartson K 1970 Is the singularity at separation removable?. J. Fluid Mech. 44, 347–364. (10.1017/S0022112070001866)

[15] Ruban AI 1982 Stability of preseparation boundary layer on the leading edge of a thin airfoil. Izv. Akad. Nauk SSSR 6, 55–63. (10.1007/BF01090379)

[16] Braun S Kluwick A 2005 Blow-up and control of marginally separated boundary layers. Phil. Trans. R. Soc. A 363, 1057–1067. (10.1098/rsta.2005.1549) 16105768

[17] Ryzhov OS Smith FT 1984 Short-length instabilities, breakdown and initial value problems in dynamic stall. Mathematika 31, 163–177. (10.1112/S0025579300012407)

[18] Scheichl S Kluwick A Braun S 2011 On higher order effects in marginally separated flows. Proc. Appl. Math. Mech. 11, 587–588. (10.1002/pamm.201110283)

[19] Aigner MJ 2012 On finite time singularities in unsteady marginally separated flows. PhD thesis, Vienna University of Technology, Austria.

[20] Aigner MJ Braun S 2012 Cauchy problems and breakdown in the theory of marginally separated flows. Proc. Appl. Math. Mech. 12, 487–488. (10.1002/pamm.201210232)

[21] Ryzhov OS Bogdanova-Ryzhova EV 2006 Instabilities in boundary-layer flows on a curved surface. J. Fluid Mech. 546, 395–432. (10.1017/S0022112005007275)

[22] Turkyilmazoglu M Ruban AI 2002 A uniformly valid asymptotic approach to the inviscid–viscous interaction theory. Stud. Appl. Math. 108, 161–185. (10.1111/1467-9590.01403)

[23] Smith FT 1979 On the non-parallel flow stability of the Blasius boundary layer. Proc. R. Soc. Lond. A 366, 91–109. (10.1098/rspa.1979.0041)

[24] Wu X 2002 Generation of sound and instability waves due to unsteady suction and injection. J. Fluid Mech. 453, 289–313. (10.1017/S0022112001006905)

